# Screening of breast cancer stem cell inhibitors using a protein kinase inhibitor library

**DOI:** 10.1186/s12935-017-0392-z

**Published:** 2017-02-13

**Authors:** Hack Sun Choi, Dal-Ah Kim, Heesung Chung, In Ho Park, Bo Hye Kim, Eok-Soo Oh, Duk-Hee Kang

**Affiliations:** 1 The Division of Nephrology, Department of Internal Medicine, Ewha Womans University School of Medicine, Seoul, 07985 Republic of Korea; 2 Department of Life Science, The Research Center for Cellular Homeostasis, Ewha Womans University, Seoul, 03760 Republic of Korea; 3 Department of Microbiology, Yonsei University College of Medicine, Seoul, 03722 Republic of Korea; 4 Division of Translational and Clinical Research II, Hematologic Malignancy Branch, National Cancer Center, Goyang-si, Gyeonggi-do 10408 Republic of Korea

**Keywords:** Cancer stem cell, Mammosphere, Protein kinase inhibitor, Breast cancer

## Abstract

**Background:**

Cancer stem cells (CSCs), a subpopulation in tumors, are known to cause drug resistance, tumor recurrence and metastasis. Based on the characteristic formation of mammospheres in in vitro conditions, the mammosphere formation assay has become an essential tool for quantifying CSC activity in breast cancer research. However, manual counting of mammospheres is a time-consuming process that is not amenable to high-throughput screening, and there are occasional inaccuracies in the process of determining the mammosphere diameter. In this study, we proposed a novel automated counting method of mammosphere using the National Institute of Standards and Technology (NIST)’s Integrated Colony Enumerator (NICE) with a screening of protein kinase library.

**Methods:**

Human breast cancer cell line MCF-7 was used for evaluation of tumor sphere efficiency, migration, and phenotype transition. Cell viability was assessed using MTT assay, and CSCs were identified by an analysis of CD44 expression and ALDEFLUOR assay using flow cytometry. Automated counting of mammosphere using NICE program was performed with a comparison to the result of manual counting. After identification of inhibitors to ameliorate CSC formation by screening a library of 79 protein kinase inhibitors using automated counting in primary, secondary and tertiary mammosphere assay, the effect of selected kinase inhibitors on migration, colony formation and epithelial-to-mesenchymal transition (EMT) of MCF-7 cells was investigated.

**Results:**

Automated counting of mammosphere using NICE program was an easy and less time-consuming process (<1 min for reading 6-well plate) which provided a comparable result with manual counting. Inhibition of calcium/calmodulin-dependent protein kinase II (CaMKII), Janus kinase-3 (JAK-3), and IκB kinase (IKK) were identified to decrease the formation of MCF-7-derived CSCs in primary, secondary and tertiary mammosphere assay. These protein kinase inhibitors alleviated TGF-β1-induced migration, colony formation and EMT of MCF-7 cells.

**Conclusions:**

We have developed a novel automated cell-based screening method which provided an easy, accurate and reproducible way for mammosphere quantification. This study is the first to show the efficacy of an automated medium-throughput mammosphere-counting method in CSC-related research with an identification of protein kinase inhibitors to ameliorate CSC formation.

## Background

Cancer stem cells (CSCs) are a highly tumorigenic cell type, comprising the minority of the tumor population. CSCs are generally defined by self-renewal characteristics and their ability to differentiate into non-stem cancer cells [1]. Another important characteristic of CSCs is the resistance to conventional chemotherapy and radiotherapy, which causes a drug-resistance and relapse [2]. Therefore, therapeutic approaches to target CSCs emerge as a promising strategy for anti-cancer treatment. Therefore, the important effort in CSC research is the identification and quantitation of CSCs in laboratory and clinical settings. In addition to cell sorting-based CSC identification method, marker-specific tracking of subpopulations of cancer cells in competitive tumor models has recently been used to further establish in vitro and in vivo evidence for the presence of CSCs. CSCs have distinct markers allowing for their isolation based on the expression of specific proteins, which includes CD44, CD133, Oct4, Sox2, Nanog, ALDH-1, ABCG, and CXCL12 [3]. Currently, the standard methods for assaying CSC activity are in vivo transplantation and in vitro tumorsphere formation [4].

Manual counting method of tumorspheres in in vitro experiment is time-consuming and requires the discrimination of spheres >50 μm in diameter by the naked eye, which makes this assay sometimes unreliable and difficult to reproduce. For accurate quantification of mammospheres, we need reliable, reproducible and convenient assay.

Understanding the mechanism of CSC formation has a paramount significance since it could help identify target genes and/or signaling pathways that are responsible for gene activation and determination of tumor microenvironment. Recent evidence suggests that cells undergoing the epithelial-to-mesenchymal transition (EMT) acquire stem cell-like characteristics [5], suggesting EMT as a potential mechanism of CSC formation.

In this study, we proposed the method of medium-throughput and automated mammosphere counting using publicly available colony counting software, the National Institute of Standards and Technology (NIST)’s Integrated Colony Enumerator (NICE) [6]. To examine the efficacy of the NICE program, we screened a library of 79 protein kinase inhibitors and validated the effect of CaMKII, JAK-3 and IκB IKK inhibitors on mammosphere formation and EMT of breast cancer cells, which suggest potential target for treatment of breast cancer via modulating the development and growth of CSC.

## Methods

### Materials

Tissue culture plates including 6- and 24-well ultra-low attachment cluster plates were obtained from Nunc Labware (Waltham, MA, USA). Protein kinase inhibitor library was purchased from Enzo Life Science (Screen-Well™ Kinase Inhibitor Library, Plymouth Meeting, PA, USA). Chemicals were obtained from Sigma-Aldrich Co. (St. Louis, MO, USA), unless otherwise stated.

### Culture of human breast cancer cells and formation of mammospheres

Human breast cancer cells, MCF-7, were obtained from the American Type Culture Collection (ATCC; Manassas, VA, USA). MCF-7 cells were grown in Dulbecco’s Modified Essential Medium (DMEM; Hyclone, Logan, UT, USA) with 10% fetal bovine serum (FBS; Hyclone), 100 U/ml penicillin, and 100 μg/ml streptomycin (Hyclone). Cells were maintained at 37 °C in a humidified incubator with 5% CO_2_. To establish primary mammospheres, MCF-7 cells were seeded at a density of 3.5–4 × 10^4^ cells/well in ultra-low attachment 6-well plates containing 2 ml of complete MammoCult™ medium (StemCell Technologies, Vancouver, BC, Canada) which was supplemented with 4 μg/ml heparin, 0.48 μg/ml hydrocortisone, 100 U/ml penicillin, and 100 μg/ml streptomycin. To establish secondary mammosphere, primary mammosphere was collected into individual conical tubes by gentle centrifugation at 1000 rpm for 1 min. After removal of supernatant, cell pellet was trypsinized with 1X EDTA/Trypsin, followed by replating a single cell suspension at a density of 4 × 10^4^ cells/well in ultra-low attachment 6-well plates containing 2 ml of complete MammoCult™ medium [7]. In 5 days, the number and size of the mammosphere were assessed compared with control. The same procedure was repeated for tertiary mammospheres [7].

### Manual counting of mammospheres

After 7-day culture of MCF-7 cell with complete MammoCult™ medium, mammospheres larger than 50 μm in diameter were manually counted under an inverted microscope (Carl Zeiss AG, Oberkochen, Germany) by two researchers (Choi HS & Kim DA) to reduce the counting bias of a single researcher. The number of mammospheres was determined from at least three independent experiments.

### Automated counting of mammospheres

MCF-7 cells were plated at a density of 4 × 10^4^ cells/well in ultra-low attachment 6-well plates containing complete MammoCult™ medium. After 7-day culture of MCF-7 cell with complete MammoCult™ medium, 8-bit gray scale image of mammospheres was acquired by placing the cell culture plate on a scanner (HP ScanjetG4050, Hewlett-Packard, Palo Alto, CA, USA). Images at high resolution (600 dpi) were loaded using the software program NICE, which was downloaded from ftp://ftp.nist.gov/pub/physics/mlclarke/NICE [6]. For counting, regions of interest (ROIs) were created by choosing the desired number of rows and columns (e.g. 2 × 3 for a 6-well plate and 4 × 6 for a 24-well plate), and individual ROIs were defined by moving and resizing the provided ROI shapes after selecting the ‘elliptical’ setting of the NICE program. The background signal of the images was negated using thresholding algorithms, and the selected images were automatically counted. To avoid counting immature mammosphere, we set the threshold parameters. Raw images were processed by background manual setting. We then set parameters using resolution and value which are set to medium and 200, respectively. The mammosphere formation assay determines the mammosphere formation efficiency (MFE, %), which corresponds to the number of mammospheres per 100× MCF-7 cells. Mammosphere size in primary mammosphere assay was measured using the software NIS-Elements BR 4.4 (Nikon, Tokyo, Japan).

### Screening of protein kinase inhibitors using automated counting method

The kinase inhibitor library containing 79 compounds (Screen-Well™ Kinase Inhibitor Library, Plymouth Meeting, PA, USA) was used to evaluate the inhibitory effect on mammosphere formation. In 24 h after an addition of MammoCult media to MCF-7 cells, cells were treated with each inhibitor at a final concentration of 10 μM. Number & size of mammosphere were assessed on day 7 of mammosphere culture.

### Flow cytometric analysis of CD44 and CD24 expression

Expression of CD44 and CD24 was determined by FACS analysis in MCF-7 cells. After harvesting and trypsinization of cells using 1X Trypsin/EDTA, one million cells were suspended and labeled with FITC-conjugated anti-human CD44 and PE-conjugated anti-human CD24 antibodies (BD Pharmingen, San Diego, CA, USA) and incubated at 4 °C for 30 min. Then, the cells were washed three times with 1X PBS and analyzed on flow cytometry (Accuri C6, BD, San Diego, CA, USA).

### ALDEFLUOR assay

CSCs were also identified by ALDEFLUOR assay (StemCell Technologies) by measuring the activity of aldehyde dehydrogenase (ALDH). MCF-7 cells were treated with 10 μM of protein kinase inhibitors for 24 h, and the proportion of ALDH-positive cells was analyzed with ALDEFLUOR assay. Briefly, MCF-7 cells were suspended with buffer containing BODIPY-aminoacetaldehyde and incubated for 45 min at 37 °C. For each sample, cells were incubated with or without diethylaminobenzaldehyde (DEAB), an ALDH inhibitor. ALDH-positive and negative cells were sorted using the FACSCalibur flow cytometer (BD Bioscience).

### Cell proliferation and viability assay

Cell proliferation and viability were assessed by CellTiter 96^®^ aqueous one solution cell proliferation assay kit (Promega, Madison, WI, USA). MCF-7 cells were grown in a 96-well plate in the presence of kinase inhibitors (5, 10 and 20 μM) with DMEM media with 10% (v/v) FBS for 48 h. The optical density was determined at 490 nm (Dynex Revelation, Dynex Ltd., Billingshurst, UK). Each series of data were determined in triplicate.

### Transwell migration assay

Each well of a transwell plate (8.0 μm pore size; Corning Costar, Tewksbury, MA, USA) was coated with gelatin B (1 μg/ml) and dried at 25 °C for 1 h, and inserted into 24-well plate which was filled with FGF2 (100 ng/ml)-containing medium. MCF-7 cells were seeded on each upper chamber at a density of 2 × 10^5^ with protein kinase inhibitors (10 μM). The plate was incubated at 37 °C in 5% CO_2_ for 24 h. Cells were stained with 0.6% hematoxylin and 0.5% eosin (H&E), and the upper surface of the filter was carefully wiped with cotton-tipped applicator. The number of cells migrating to the lower surface of the filter was counted.

### Colony formation assay

7 × 10^4^ of MCF-7 cells were plated in each well of 6-well plates with 0.3% top agar over a 0.6% agar layer. Protein kinase inhibitors were added to the top agar at a concentration of 10 μM. Dishes were incubated for 7 days until colonies were large enough to be visualized. Colonies were stained with 0.005% crystal violet for 1 h, and the number of clones was counted.

### Western blotting

Proteins isolated from MCF-7 cell or MCF-7-derived mammospheres treated with protein kinase inhibitors were separated on 10% SDS-PAGE and transferred to a polyvinylidine difluoride membrane (Millipore, Bedford, MA, USA). Membranes were blocked in 5% nonfat dry milk in PBS-Tween 20 (0.1%, v/v) at room temperature for 30 min. The blots were incubated in blocking solution with primary antibodies at 4 °C overnight. Primary antibodies used in this experiment were following: mouse anti-cytokeratin-18, (CK18) (Santa Cruz, CA, USA), rabbit anti-CD24 (Santa Cruz, CA, USA), mouse anti-β-actin (Santa Cruz, CA, USA), mouse anti-N-cadherin (BD Bioscience, Bedford, MA, USA), and mouse anti-CD44 (Cell signaling, Danvers, MA, USA). After washing with PBS-Tween 20 (0.1%, v/v), the blots were incubated with horseradish peroxidase (HRP)-conjugated secondary antibodies corresponding each primary antibodies and enhanced using the chemiluminescence detection (Santa Cruz).

### Statistical analysis

All data were presented as the mean ± standard deviation (SD). Data were analyzed using Student’s *t* test. A p value lower than 0.05 was considered statistically significant (GraphPad Prism 5 Software, San Diego, CA, USA).

## Results

### Formation of mammospheres from MCF-7 cells

**Fig. 1:**
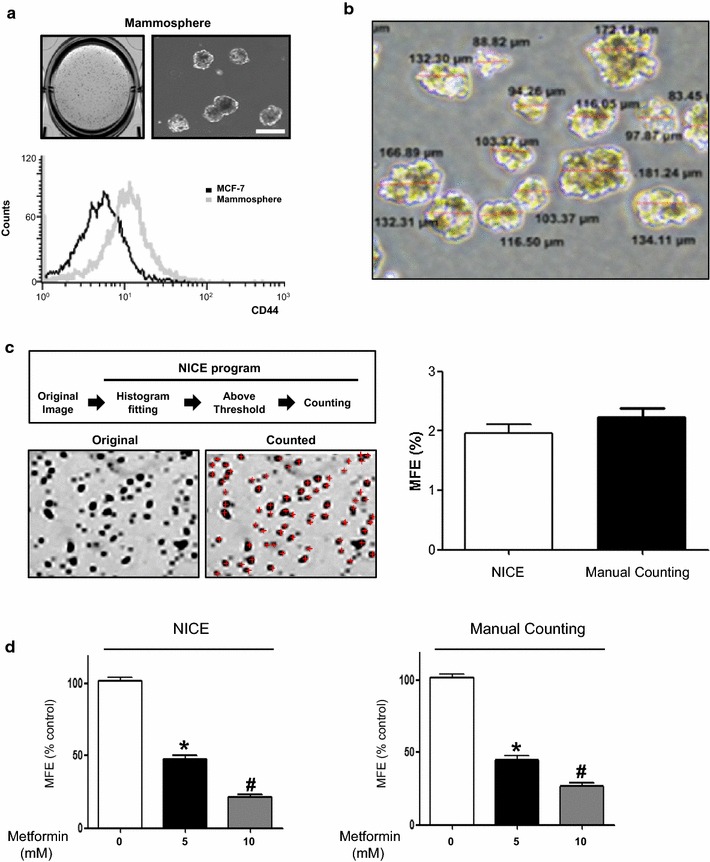
Formation of mammospheres from MCF-7 cells and automated counting of mammospheres using NICE software. **a** Spheres derived from a single cell suspension of MCF-7 cells are visible in 7 days of culture with complete MammoCult™ medium. CD44 expression in mammospheres (*gray*) assessed by FACS analysis using FITC-anti-CD44 is higher compared to adherent MCF-7 cells (*black*). **b** The representative image of tumor spheres are shown with diameter of each measured by NIS-Elements BR4.4 software (Nikon, Tokyo, Japan). **c** Among mammospheres in the initial scanned image (Original), spheres larger than 50 μm in diameter are selected, counted and marked with a red crosshair (Counted) after histogram pitting using NICE software. The number of mammospheres counted by NICE software is comparable with the result of manual counting by naked eye under an inverted microscope. **d** Metformin (5 and 10 mM) significantly ameliorates the formation of mammospheres, which is demonstrated as a decrease in mammosphere formation efficiency (MFE) quantified by the NICE program and manual counting. The data shown are the mean ± SD of 3 independent experiments. *p < 0.05 vs. 0 mM. ^#^p < 0.05 vs. 0 and 5 mM. The *scale bar* is 100 μm

An incubation of MCF-7 cells with complete MammoCult™ medium resulted in the formation of spherical mammospheres in 7 days (Fig. 1a). Mammospheres derived from MCF-7 cells in suspension culture harbored higher CD44 levels compared to adherent MCF-7 cells (Fig. 1a). Mean diameter of mammosphere was 134.7 μm (range 80–230 μm) in 7 days of mammosphere culture (Fig. 1b).

### Automated counting of MCF-7 mammospheres using the NICE program

Automated mammosphere counting using NICE program was less time-consuming (<1 min for reading 6-well plate) compared to manual counting with an easy exclusion of immature mammosphere less than 50 μm. Mammosphere formation efficiency (MFE) of MCF-7 cells assessed by automatic and manual counting were comparable (1.96 vs. 2.23%, p > 0.05) (Fig. 1c). To confirm whether automated counting assay was on a par with manual counting, we examined the effect of metformin (5 and 10 mM), an antidiabetic drug known to inhibit mammosphere formation [8]. An inhibitory effect of metformin on MFE assessed by automated and manual counting was comparable (Fig. 1d).

### Screening of the effect of protein kinase inhibitors on mammosphere formation

**Fig. 2:**
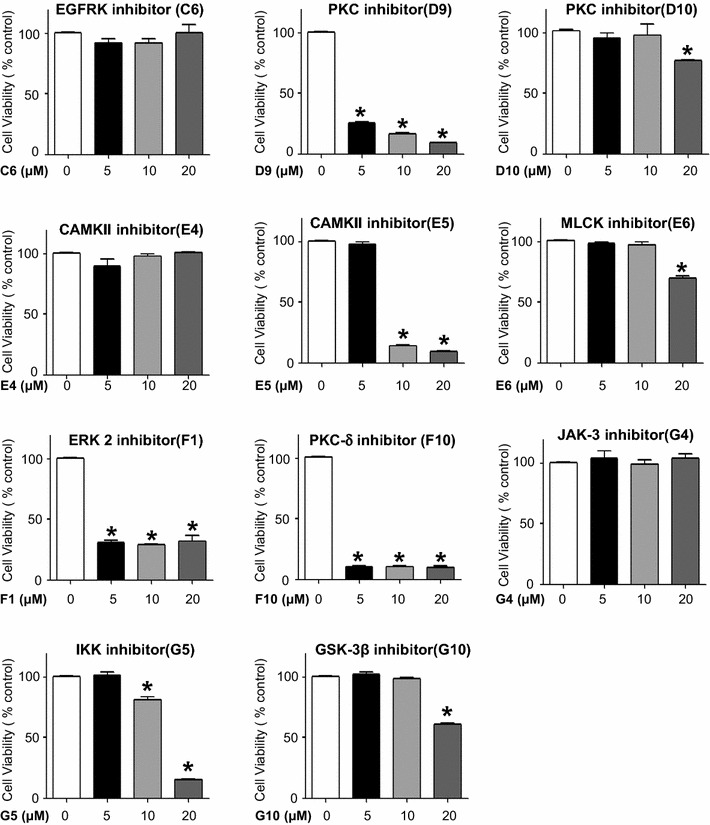
Effects of protein kinase inhibitors on the viability of MCF-7 cells. Among 11 protein kinase inhibitors selected based on an inhibitory effect on mammosphere formation using the NICE program, the PKC inhibitor (D9), CAMKII inhibitor (E5), ERK 2 inhibitor (F1), and PKC-δ inhibitor (F10) significantly decrease cell viability at 48 h. The data shown represent the mean ± SD of 3 independent experiments. *p < 0.05 vs. each control

In order to find the protein kinase inhibitors possessing CSC-suppressing effect, we screened protein kinase library consisting of 79 compounds (Screen-Well™ Kinase Inhibitor Library, Plymouth Meeting, PA, USA) by automatic counting using NICE program. As a result, we identified 11 protein kinase inhibitors with MFE by more than 50% inhibition at a final concentration of 10 µM (Table 1). They were specific for Epidermal Growth Factor Receptor Kinase (EGFRK) (C6), PKC (D9 and D10), CaMKII (E4 and E5), Myosin Light Chain Kinase (MLCK) (E6), Extracellular Regulated Kinase 2 (ERK2) (F1), Protein Kinase C delta (PKC δ) (F10), JAK-3 (G4), IκB Kinase (IKK) (G5), and GSK-3β (G10). After assessing the cytotoxicity of these inhibitors on MCF-7 cells, we excluded 4 kinase inhibitors that significantly decreased the viability of MCF-7 cells at a concentration of 10 μM (Fig. 2). Finally, 7 kinase inhibitors were selected as potential suppressors of breast CSC formation, which were specific for EGFRK, PKC, CaMKII, MLCK, JAK, IKK, and GSK-3β (Fig. 2).

**Table 1:** Summary of protein kinase inhibitors suppressing mammosphere formation

Number	Name	Targets	Inhibitory activity	Number	Name	Targets	Inhibitory activity
B1	PD-98059	MEK	Negative	C1	Tyrphostin 46	EGFRK, PDGFRK	Negative
B2	U-0126	MEK	Negative	C2	Tyrphostin 47	EGFRK	Negative
B3	SB-203580	p38 MAPK	Negative	C3	Tyrphostin 51	EGFRK	Negative
B4	H-7 2HCl	PKA, PKG, MLCK, PKC	Negative	C4	Tyrphostin 1	Negative control for tyrosine kinase	Negative
B5	H-9 HCl	PKA, PKG, MLCK, PKC	Negative	C5	Tyrphostin AG 1288	Tyrosine kinases	Negative
B6	Staurosporine	Pan-specific	Negative	C6	Tyrphostin AG 1478	EGFRK	Positive
B7	AG-494	EGFRK, PDGFRK	Negative	C7	Tyrphostin AG1295	Tyrosine kinases	Negative
B8	AG-825	HER1-2	Negative	C8	Tyrphostin 9	PDGFRK	Negative
B9	Lavendustin A	EGFRK	Negative	C9	Hydroxy-2-naphthalenyl methylphosphonic acid	IRK	Negative
B10	RG-1462	EGFRK	Negative	C10	PKC-412	PKC inhibitor	Negative
B11	Tyrphostin 23	EGFRK	Negative	C11	Piceatannol	Syk	negative
B12	Tyrphostin 25	EGFRK	Negative	C12	PP1	Src family	Negative
D1	AG-490	JAK-2	Negative	E1	HA-1004 2HCl	PKA, PKG	Negative
D2	AG-126	IRAK	Negative	E2	HA-1077 2HCl	PKA, PKG	Negative
D3	AG-370	PDGFRK	Negative	E3	2-Hydroxy-5-(2,5-dihydroxy benzyl amino) benzoic acid	EGFRK, CaMK II	Negative
D4	AG-879	NGFRK	Negative	E4	KN-62	CaMK II	Positive
D5	LY 294002	PI 3-K	Negative	E5	KN-93	CaMK II	Positive
D6	Wortmannin	PI 3-K	Negative	E6	ML-7 HCl	MLCK	Positive
D7	GF 109203X	PKC	Negative	E7	ML-9 HCl	MLCK	Negative
D8	Hypericin	PKC	Negative	E8	2-Aminopurine	p58 PITSLRE beta1	Negative
D9	Ro 31-8220 mesylate	PKC	Positive	E9	N9-isopropyl-olomoucine	CDK	Negative
D10	d-Erythro-Sphingosine	PKC	Positive	E10	Olomoucine	CDK	Negative
D11	H-89 2HCl	PKA,	Negative	E11	Iso-olomoucine	Negative control for olomoucine	Negative
D12	H-8	PKA, PKG	Negative	E12	Roscovitine	CDK	Negative
F1	5-Iodotubericidin	ERK2, CK1, CK2	Positive	G1	Erbstatin analog	EGFRK	Negative
F2	LFM-A13	BTK	Negative	G2	Quercetin 2H2O	PI 3-K	Negative
F3	SB-202190	p38 MAPK	Negative	G3	SU1498	Flk1	Negative
F4	PP2	Src family	Negative	G4	ZM 449829	JAK-3	Positive
F5	ZM 336372	cRAF	Negative	G5	BAY 11-7082	IKK pathway	Positive
F6	SU 4312	Flk1	Negative	G6	5,6-dichloro-1-β-d-ribofuranosylbenzimidazole	CK II	Negative
F7	AG-1296	PDGFRK	Negative	G7	2,2′,3,3′,4,4′-Hexahydroxy-1,1′-biphenyl-6,6′dimethanol dimethyl ether	PKC alpha, PKC gamma	Negative
F8	GW 5074	cRAF	Negative	G8	SP 600125	JNK	Negative
F9	Palmitoyl-d l-carnitine	PKC	Negative	G9	Indirubin	GSK-3beta, CDK5	Negative
F10	Rottlerin	PKC delta	Positive	G10	Indirubin-3′-monooxime	GSK-3beta	Positive
F11	Genistein	Tyrosine kinases	Negative	G11	Y-27632 2HCl	ROCK	Negative
F12	Daidzein	Negative control for genistein	Negative	G12	Kenpaullone	GSK-3beta	Negative
H2	Triciribine	Akt signaling	Negative	H6	Apigenin	CK-II	Negative
H3	BML-257	Akt	Negative	H7	BML-265	EGFRK	Negative
H4	SC-514	IKK2	Negative	H8	Rapamycin	mTOR	Negative
H5	BML-259	Cdk5/p25	Negative

### Validation of the inhibitory effects of protein kinase inhibitors on mammosphere formation

**Fig. 3:**
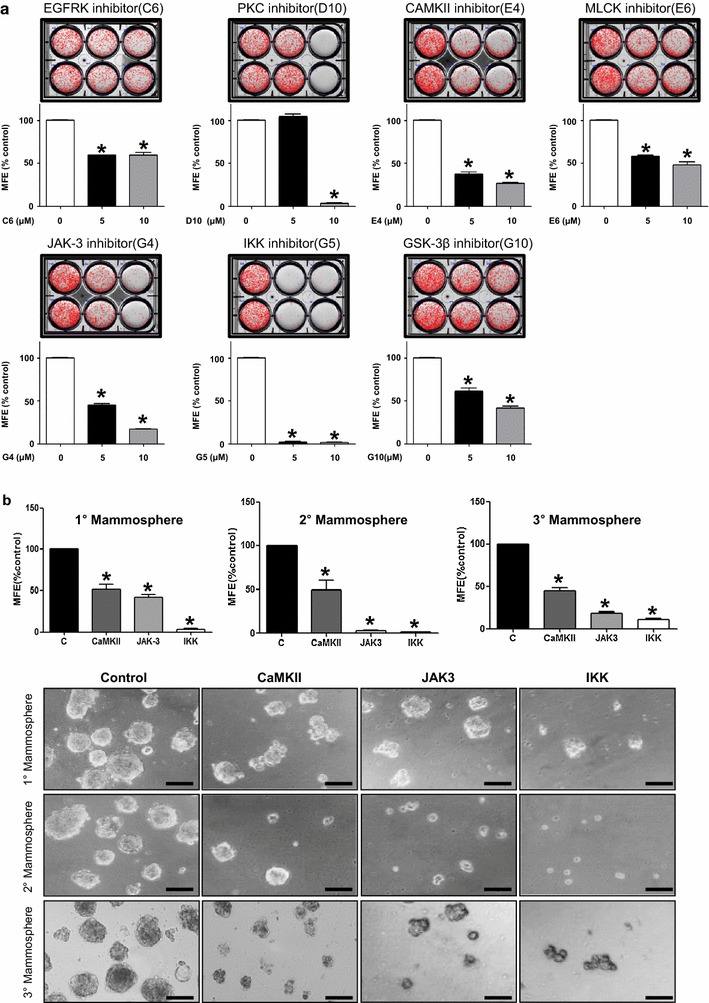
Effects of protein kinase inhibitors on the mammosphere formation. **a** EGFRK, PKC, CAMKII, MLCK, JAK-3, IKK, and GSK-3β inhibitors significantly decrease the mammosphere-forming efficiency (MFE) at the concentrations of 5 or 10 μM. In particular, the CAMKII (E4), JAK-3 (G4), and IKK (G5) inhibitors demonstrate >50% inhibition of MFE at 5 μM. **b** The inhibitory effect of CAMKII (E4), JAK-3 (G4), and IKK (G5) inhibitors on MFE is also observed in secondary and tertiary mammosphere assay. Representative images with quantitation bar are shown (*scale bar* 100 μm). *p < 0.05 vs. each control

We further analyzed whether these 7 protein kinase inhibitors suppressed the formation of mammospheres at a lower concentration (5 μM) by automatic counting. Inhibitors specific for CaMKII (E4), JAK-3 (G4), and IKK pathway (G5) demonstrated a significant inhibitory effect on mammosphere formation by more than 50% (Fig. 3a). Furthermore, an inhibitory effect of these protein kinase inhibitors (E4, G4, and G5) was observed in secondary and tertiary mammosphere assay (Fig. 3b). Not only the number, but the size of mammosphere was also decreased with co-treatment of CaMKII, JAK3 and IKK inhibitors by 29.9, 54.2, 36.9%, respectively.

### Effect of CaMKII, JAK-3, and IKK inhibitors on the proportion of ALDH-positive breast cancer cells and CD44^high^/CD24^low^-expressing subpopulation

**Fig. 4:**
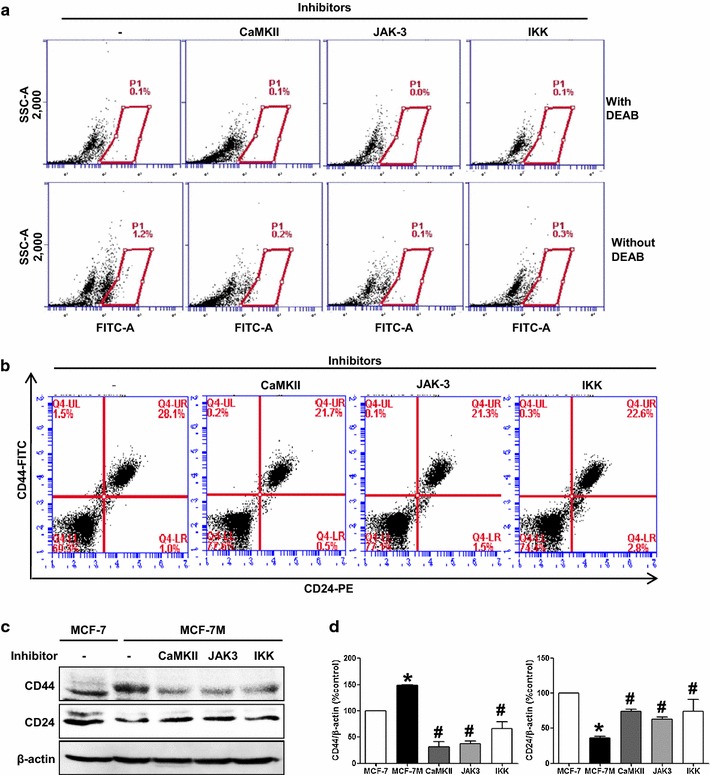
Effects of protein kinase inhibitors on the expression of ALDH, CD44 and CD24.** a** ALDH-positive cells, quantified by the ALDEFLUOR assay, are significantly decreased with treatment of CAMKII, JAK-3, and IKK inhibitors (10 µM) in MCF-7 cells. The *upper panel* shows ALDH-positive cells which are treated with ALDH inhibitor, DEAB, as a negative control, and the *lower panel* represents ALDH-positive cells without DEAB. The ALDH-positive population is gated as demonstrated in *box*.** b** The proportion of CD44^high^/CD24^low^ cell population is further analyzed by flow cytometry in MCF-7 cells with a treatment of CAMKII, JAK-3, and IKK inhibitors or DMSO for 2 days.** c**,** d** The effect of CaMKII, JAK3, IKK inhibitors on the expression of CD44/CD24 in MCF-7-derived mammosphere (MCF-7 M) is also demonstrated in western blotting. β-actin is used as a loading control. Representative western blot analysis with a quantitation *bar* is shown. The data shown represent the mean ± SD of 3 independent experiments. *p < 0.05 vs. MCF-7, ^#^p < 0.05 vs. MCF-7 M

To further confirm the role of CaMKII, JAK-3, and IKK kinase in CSC formation, we investigated the effect of these kinase inhibitors on proportion of breast cancer cells with ALDH activity and CD44^high^/CD24^low^-expressing subpopulation in MCF-7 cells. CaMKII, JAK-3, and IKK inhibitors decreased the proportion of ALDH-positive breast cancer cells (Fig. 4a) and CD44^high^/CD24^low^ subpopulation (Fig. 4b). The CD44 expression of MCF-7-derived mammosphere was higher than MCF-7 cells, suggesting the stemness of mammosphere. CaMKII, JAK3 and IKK inhibitors decreased CD44 expression with an increase in CD24 in mammosphere (Fig. 4c, d).

### Effect of CaMKII, JAK-3, and IKK inhibitors on migration, colony formation, and EMT of MCF-7 cells

**Fig. 5:**
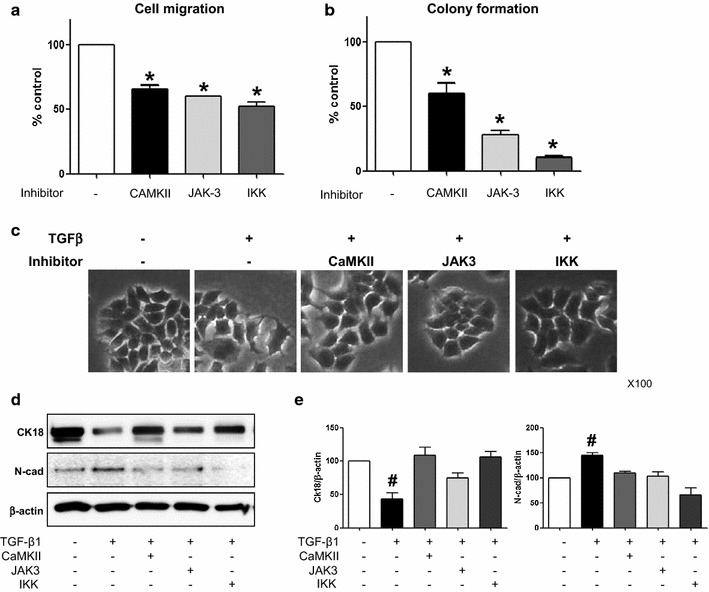
Effects of protein kinase inhibitors on migration, colony formation ability and TGF-β1-induced EMT in MCF-7 cells.** a**,** b** The inhibitors of CAMKII, JAK-3 and IKK decrease cell migration (**a**) and colony formation (**b**) in MCF-7 cells.** c** TGF-β1 (10 ng/ml) induces morphological changes in MCF-7 cells, including a loss of cell contact and elongation, which are ameliorated by CAMKII, JAK-3 and IKK inhibitors (10, 10, 5 µM, respectively). Representative photos of cell morphology are shown (×100 magnification).** d**,** e** Western blot analysis with a quantitation bar demonstrates TGF-β1-induced EMT as a decrease in an epithelial cell marker, cytokeratin 18 (CK18), and an increase in a mesenchymal marker, N-cadherin (N-cad). TGF-β1-induced EMT is ameliorated by CAMKII, JAK-3 and IKK inhibitors. β-actin is used as a loading control. The data shown represent the mean ± SD of 5 independent experiments. *p < 0.05 vs. control, ^#^p < 0.05 vs. others

The CaMKII, JAK-3, and IKK inhibitors decreased the migration and colony formation of MCF-7 cells (Fig. 5a, b). In addition, these inhibitors ameliorated the TGF-β1-induced EMT in MCF-7 cells (Fig. 5c, d). As shown in Fig. 5c, TGF-β1 induced a morphologic transition of MCF-7 cells including an elongation of cell shape with a loss of cell-to-cell contact, which was alleviated by CaMKII, JAK-3, and IKK inhibitors. CaMKII, JAK-3, and IKK inhibitors also reversed TGF-β1-induced alteration of the expression of epithelial and mesenchymal cell markers, CK18 and N-cadherin, respectively (Fig. 5d, e).

## Discussion

In this study, we proposed an easy, accurate and less time-consuming method of tumor sphere quantitation using NICE program for the first time. Using this automatic counting method, we screened the protein kinase inhibitor library, and identified 3 protein kinase inhibitors, which had a significant inhibitory effect on formation of primary, secondary and tertiary mammosphere of breast cancer cells. We further confirmed the effect of these protein kinase inhibitors targeting CaMKII, JAK-3, and IKK on cell migration, colony formation and TGF-β-induced EMT of MCF-7 cells.

Human solid tumors are phenotypically and functionally heterogeneous. Solid tumors contain CSCs that are responsible for sustaining tumor growth, which can partially account for the functional heterogeneity of solid cancer. CSCs have the capacity for self-renewal and generating tumors resembling the parental tumors, which can be a cause of drug resistance or recurrence of cancer [3]. In breast cancer, CSCs have been identified as a subpopulation of tumor [9, 10]. The recurrence rate of breast cancer is approximately 40%, and the recurrence is known to be closely linked to patients’ survival. Therefore, the research on CSCs is one of the most promising ways to improve patients’ survival with an understanding of the mechanisms by which CSCs are developed and maintained.

The gold standard assay for studying CSCs is xenograft transplantation, however a simple and cost-effective assay for examining CSC activity is tumorsphere formation assay [11]. The mammosphere formation assay in breast cancer is based on the special property of CSCs to survive and grow in an anchorage-independent manner, while differentiated cancer cells undergo anoikis or die [12]. Although it is challenging to isolate and enrich the CSCs from a solid tumor, culturing of stem cells from breast cancer tissue or cell lines has been well adapted in breast cancer research [10, 13].

In this study, we proposed the method of automated mammosphere counting, which allowed for medium-throughput screening of small molecules or chemicals as potential inhibitors of CSCs activity. We confirmed the automated counting using the NICE program is reliable and more convenient compared to manual counting. The mammosphere counting procedure using the NICE program suggested in this study consists of the following three steps: (1) acquiring an original image, (2) operating the NICE program, and 3) counting the mammospheres which took less than 1 min for reading 6-well plate. In general, manual counting is a time-consuming process and discriminating mammospheres larger than 50 μm in diameter by naked eyes can be difficult, resulting in potentially inaccurate data. The automated counting method proposed by this study can overcome these limitations of manual counting currently in use. Recently, Choudhry P also reported the accuracy and reliability of colony counting using several open-source digital methods, which demonstrated Cell Colony Edge Detection using ImageJ matched the best with manual counting with superior results compared to other open-source methods in speed, accuracy and applicability [14]. Although automated counting using NICE program was not included in Choudhry P’s analysis with no direct comparison data, each method seems to have a unique strength and limitation for colony counting in specific experimental condition and the researchers have more choices for automatic measurement of tumor spheroid.

Conventional anti-cancer drugs with a proven killing effect on non-tumorigenic cancer cells usually fail to effectively eliminate CSCs [15], which is one of the causes of tumor recurrence. To identify new anti-cancer drugs against CSCs, it is crucial to understand the cellular events involved in the development and survival of CSC. Targets against CSCs such as surface markers, telomerase, CSC niche proteins, drug transporter proteins, and differentiation-related proteins are proposed [16], however the most robust targets for killing CSCs are proteins involved in signaling pathways [16]. To find novel inhibitors of CSC formation, we have screened a protein kinase inhibitor library containing 79 chemicals that target the following kinases: CaMK, CDK, CKI & II, EGFRK, GSK, IKK, insulin receptor, JAK, JNK, MAPK, MEK, MLCK, PI3K, PDGFRK, PKA, PKC, RAF, SAPK, Src-family kinases, VEGFR and others. Among them, compounds specifically inhibiting mammosphere formation without affecting the growth of differentiated cancer cells were first selected. Finally, we identified 3 compounds that effectively inhibited the formation of mammospheres at lower concentration. Since Studies have shown that using primary spheroid inhibition alone might not necessarily identify agents targeting true self-renewing CSCs [7], we further validated the effect of the inhibitors of CaMKII, JAK-3 and IKK in subsequent passage of primary spheroid-derived cells into secondary and tertiary spheroid condition.

KN-62 (E4) is a cell-permeable CaMKII inhibitor. KN-62 has been shown to effectively suppress the expression of HIF-1α in hepatoma cells [17]. ZM 449829 (G4) is a selective JAK-3 inhibitor. STAT3, a downstream target of JAK-3, is required for the proliferation and self-renewal of cancer stem cells [18–21]. BAY 11-7082 (G5), an IKK inhibitor, is reported to repress mammosphere formation. Inhibitors of NF-κB pathway have previously been shown to inhibit breast cancer stem cell-like cells [22]. In addition, it was reported that BAY 11-7082 induced apoptosis and cell cycle arrest in gastric cancer [23].

Our data showed that the selected kinase inhibitors which suppressed CSC formation partially alleviated EMT of breast cancer cells. The main signaling pathway in TGF-β-induced EMT is the Smad pathway [24]. One of the major mechanisms of CSC formation is a phenotype transition of cancer cells, such as EMT. The process of EMT is characterized by the loss of epithelial cell markers with an overexpression of mesenchymal markers [5, 9, 25]. The kinase inhibitors evaluated in this study ameliorated TGF-β-induced increase in N-cadherin expression and inhibited the migration and colony formation ability of breast cancer cells. Further studies are necessary to elucidate the molecular mechanism of anti-CSC effects of CaMKII, JAK-3 and IKK inhibitors in breast cancer with a validation in animal model of breast cancer.

In conclusion, our study suggests a simple, medium-throughput, and automated mammosphere-counting method as a useful tool for CSC-related research. Furthermore, we used this assay to screen kinase inhibitors that suppress the development of breast CSC. We anticipate that our methodology can be adopted as a reliable, reproducible and easy tool for screening potential compounds targeting CSCs.

## Conclusions

We proposed automated mammosphere-counting method as a useful tool for CSC-related research with a validation of the inhibitory effect of 3 protein kinase inhibitors (CaMKII, JAK3, and IKK inhibitors) on CSC formation.

## Abbreviations

CSCs: cancer stem cells
CaMKII: calcium/calmodulin-dependent protein kinase II
JAK-3: Janus kinase-3
IKK: IκB kinase
CK18: cytokeratin 18
N-cad: N-cadherin
TGF-β1: transforming growth factor-β1
EGFRK: epidermal growth factor receptor kinase
PKC: protein kinase C
ERK: extracellular regulated kinase
MLCK: myosin light chain kinase
